# Negative Refraction of Weyl Phonons at Twin Quartz Interfaces

**DOI:** 10.1021/acsmaterialslett.3c00846

**Published:** 2024-02-05

**Authors:** Gunnar F. Lange, Juan D. F. Pottecher, Cameron Robey, Bartomeu Monserrat, Bo Peng

**Affiliations:** † Theory of Condensed Matter Group, Cavendish Laboratory, University of Cambridge, J. J. Thomson Avenue, Cambridge CB3 0HE, United Kingdom; ‡ St. Catharine’s College, University of Cambridge, Trumpington Street, Cambridge CB2 1RL, United Kingdom; ¶ St. John’s College, University of Cambridge, St John’s Street, Cambridge CB2 1TP, United Kingdom; § Department of Materials Science and Metallurgy, University of Cambridge, 27 Charles Babbage Road, Cambridge CB3 0FS, United Kingdom

## Abstract

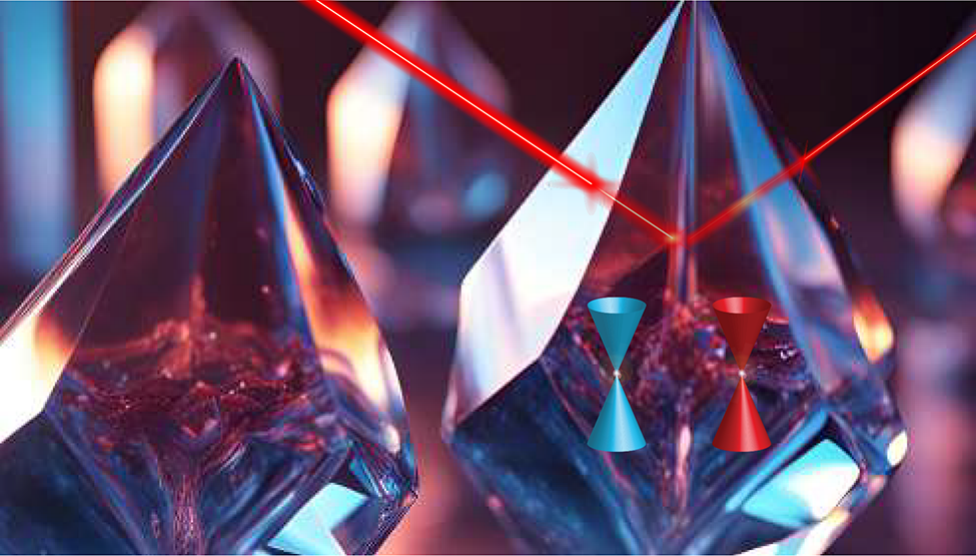

In Nature, α-quartz crystals frequently form contact twins, which are two adjacent crystals with the same chemical structure but different crystallographic orientation, sharing a common lattice plane. As α-quartz crystallizes in a chiral space group, such twinning can occur between enantiomorphs with the same handedness or with opposite handedness. Here, we use first-principles methods to investigate the effect of twinning and chirality on the bulk and surface phonon spectra, as well as on the topological properties of phonons in α-quartz. We demonstrate that, even though the dispersion appears identical for all twins along all high-symmetry lines and at all high-symmetry points in the Brillouin zone, the dispersions can be distinct at generic momenta for some twin structures. Furthermore, when the twinning occurs between different enantiomorphs, the charges of all Weyl nodal points flip, which leads to mirror symmetric isofrequency contours of the surface arcs on certain surfaces. We show that this allows negative refraction to occur at interfaces between certain twins of α-quartz.

Negative refraction is a counterintuitive phenomenon in which incident and refracted waves emerge on the same side of the interface normal,^1^ providing potential applications in superlens and subwavelength imaging.^2^ One prominent strategy to obtain negative refraction uses open isofrequency contours, which have been realized in hyperbolic metamaterials.^3−6^ Recent advances in topological materials offer a new platform to manipulate isofrequency contours arising from topological surface states.^7−16^ For example, the surface arcs of Weyl points can form distinct isofrequency contours for both positive and negative refraction,^17,18^ and all-angle reflectionless negative refraction has been observed in Weyl metamaterials.^19^ Intuitively, negative refraction can take place at the interface between chiral crystals with left- and right-handed screw symmetries, as the isofrequency contours of the surface Weyl arcs are mirror images of each other for specific choices of surface termination.

One of the most well-known chiral crystal is α-quartz, which exists in nature in two enantiomorphs that belong to a space-group pair and are thus-handed: the right-handed screw *P*3_1_21 (No. 152) and the left-handed screw *P*3_2_21 (No. 154). The crystal structures have opposite chirality, which can be distinguished either by measuring their optical activity (the rotation of the plane of polarization of plane-polarized light), as first observed in quartz crystals in 1811 by François Arago,^20,21^ or by circularly polarized resonant X-ray diffraction (XRD).^22−24^ As α-quartz is an insulator under ambient conditions, any potential electronic topology away from the Fermi level is not easily accessible. To remedy this, we instead consider the topology of the intrinsic lattice vibrations (phonons) in α-quartz, which are not constrained by the Fermi level. The topological properties of phonons have been studied extensively.^25−49^ In contrast to metamaterials, phonons are intrinsic quasiparticles in real materials that are similar to electrons. Additionally, typical phonon frequencies are in the 0–50 THz range, and negative refraction in the terahertz frequency range has been well-studied experimentally.^5^ Therefore, it is expected that negative refraction can be straightforwardly measured in topological phonons using the existing apparatus. Phonon modes in the two enantiomorphs exhibit chiral behaviors such as opposite pseudoangular momenta, selective optical transitions and opposite transport direction.^50−57^ Furthermore, band crossings between modes in a single enantiomorph of α-quartz form Weyl points because of the lack of inversion symmetry in the space groups *P*3_1_21 or *P*3_2_21,^35^ and it is well-known that the Weyl points of two enantiomorphs carry opposite Chern numbers.^58−63,63^ It is therefore expected that the Weyl phonons in α-quartz with left- and right-handed screws carry opposite Chern numbers.

In Nature, quartz naturally forms contact twin structures, two crystals with the same chemical composition but different crystallographic orientations, touching along a common plane. Twinned quartz crystals are much more common than untwinned ones on Earth,^64^ and quartz crystals are therefore generally racemic.^65,66^ In twinned crystals, many different structures with various orientation of the unit cells are possible and have been generally classified by twinning laws.^67^ As such, twinned quartz crystals offer a natural and versatile platform to study the interplay between chirality and topology, as the twinning boundaries of α-quartz should be easily accessible. The relationship between chirality and topology is a very active area of research,^61,68−78^ as chiral space groups can host a plethora of interesting topological phenomena. Hence, a careful comparison of enantiomorphic structures is of significant current interest with the potential application of negative refraction occurring at the twin boundary in α-quartz.

In this work, we explore the relationship between the twinning type of the chiral crystal structures, their phonon band structure, and their associated Weyl points. We show that, for the three most common types of quartz contact twinning, the bulk phonon band structures coincide along all high-symmetry lines in the Brillouin zone. However, depending on the twinning choice, the band structures differ at generic momenta. Furthermore, even when the bulk band structure agrees for enantiomorphic twins, the surface isofrequency contours differ. This can allow negative refraction to occur, although the details of this will depend on the nature of the twin interface. We find negative refraction for both idealized and realistic surface terminations, though the direct mapping to topology and chirality is obscured by the details of the interface in the latter case.

## Methodology

Density functional theory (DFT) calculations are carried out using the Vienna *ab initio* simulation package (vasp).^79,80^ The generalized gradient approximation (GGA) calculations are performed using the Perdew–Burke–Ernzerhof exchange-correlation functional as revised for solids (PBEsol),^81^ with four valence electrons (3*s*^2^3*p*^2^) for Si atoms and six valence electrons (2*s*^2^2*p*^4^) for O atoms. The plane-wave basis set has an upper kinetic energy limit of 800 eV and the **k**-mesh has a size of 7 × 7 × 7, with the self-consistent field loop stopped when energy differences between steps are below 10^–6^ eV. Structural relaxations are carried out until the Hellman–Feynman forces are <10^–2^ eV/Å.

Density functional perturbation theory is used in calculating Hessian matrices and phonon frequencies,^82,83^ implemented on a 3 × 3 × 2 supercell with a 3 × 3 × 3 **k**-mesh. phonopy is used to build the matrix of force constants, diagonalize the dynamical matrix, and obtain the phonon dispersion curves.^84,85^ Convergence of the calculations is assessed by varying both the supercell and **k**-mesh sizes and noting no discrepant results. The calculations include the splitting between transverse and longitudinal optical phonon modes (LO-TO splitting),^86^ but we note that LO-TO splitting plays a minor role on the topological properties of phonons away from the Brillouin zone center. WannierTools is used to locate every single band crossing point on a phonon **q**-mesh of size 51 × 51 × 51, to calculate the chiralities of the Weyl nodes, and to compute the phonon surface states via the surface Green’s function.^87^

## Crystal Structures and Lattice Dynamics

**Figure 1:**
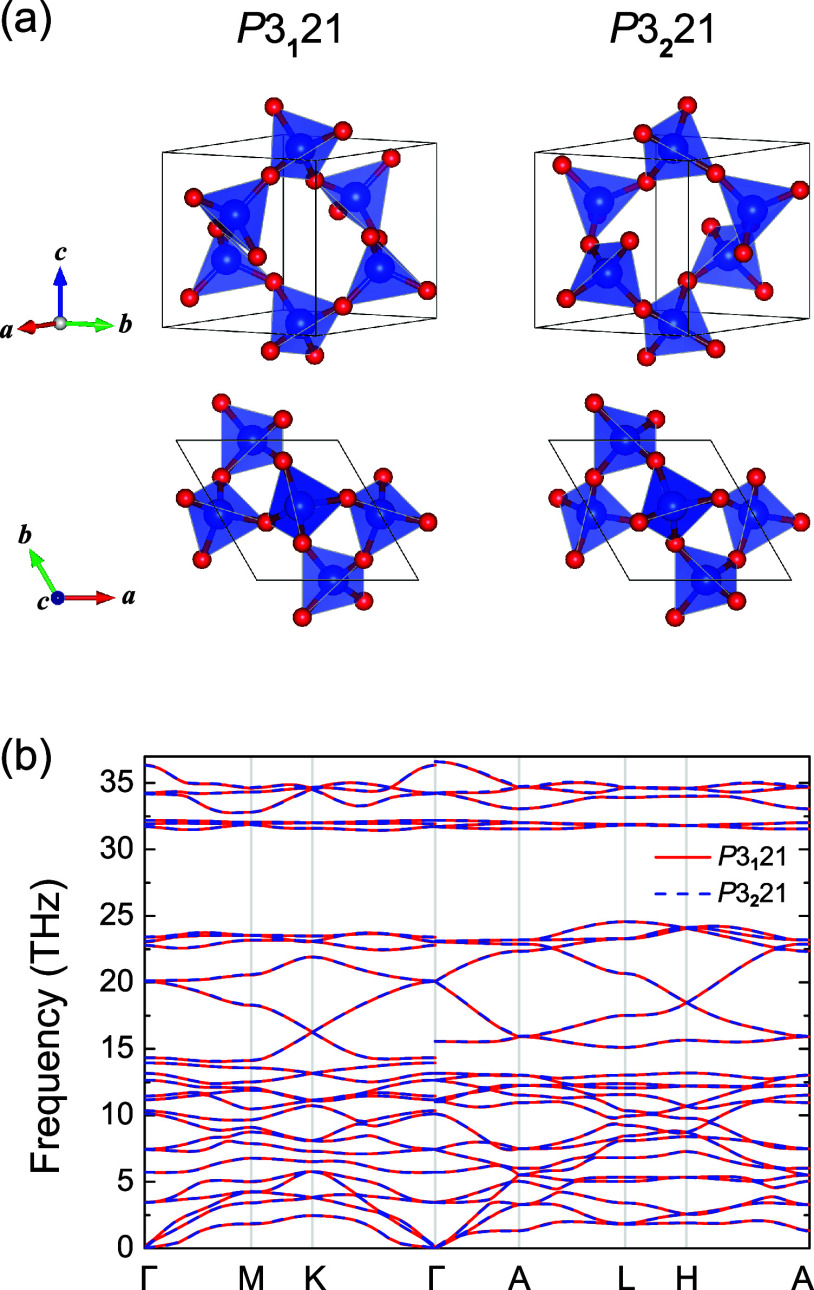
(a) Crystal structures of α-quartz with space group *P*3_1_21 (No. 152) and *P*3_2_21 (No. 154). (b) Phonon dispersion of α-quartz. The two structures are related by a mirror operation, leading to Leydolt twinning, as explored below.

**Figure 2:**
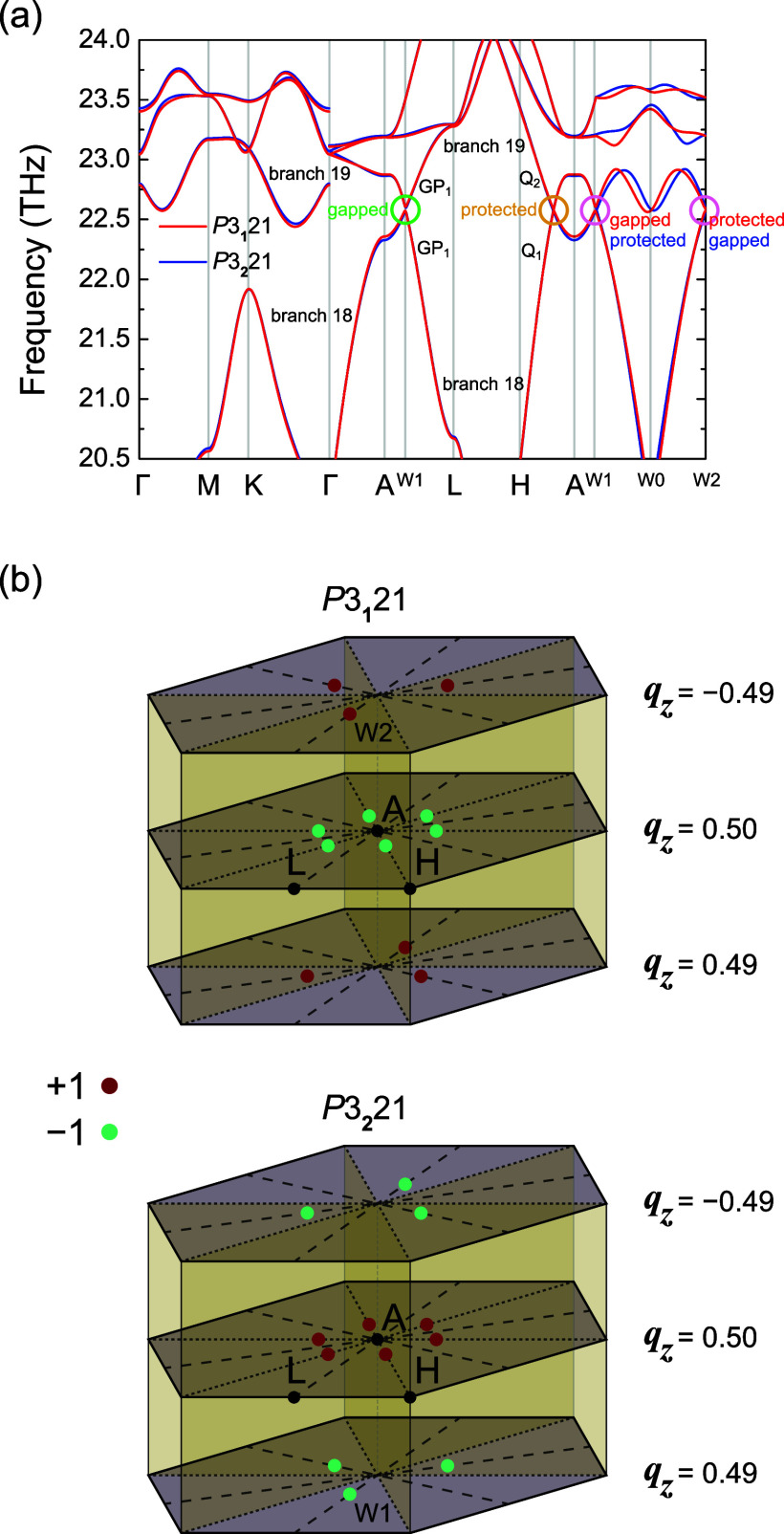
(a) Phonon branches 18 and 19 along the high-symmetry lines and along the line W1 (0.15, 0, 0.5)–W0 (0.15, 0, 0)–W2 (0.15, 0, −0.5) with a trivial unitary point group. (b) Positions of the band crossing points between branches 18 and 19 in the Brillouin zone of *P*3_1_21 (No. 152) and *P*3_2_21 (No. 154) α-quartz, with unit cells related by a mirror operation (Leydolt twinning).

α-Quartz is the most stable phase of silica under ambient conditions, and it crystallizes in the trigonal crystal system with space group *P*3_1_21 (No. 152) or *P*3_2_21 (No. 154), depending on the chirality. As shown in Figure 1a, α-quartz is composed of oxygen tetrahedra with Si atoms placed at their centers. The tetrahedra are joined at their vertices, giving two possible chiral structures. The computed lattice constants are *a* = *b* = 4.965 Å and *c* = 5.455 Å, which are in good agreement with previous measured and calculated data.^24,88−90^ We first focus on the conventional unit cell choice, where the (*x*,*y*) position of all atoms in the unit cell agree, and the difference between the enantiomorphs is solely determined by the relative atomic *z* coordinates, i.e., the enantiomorphs are related by a mirror symmetry, *m*_*z*_, with respect to the *z* = 0 plane. This corresponds to Leydolt twinning, as explored below. Using this unit cell, we show the phonon spectra for α-quartz with both space groups in Figure 1b, agreeing well with previous calculations and measurements.^35,90−93^ No imaginary mode is found in the entire Brillouin zone, indicating their dynamic stability. For this choice of twinning, we find that the phonon dispersions of the two chiral structures agree along all high-symmetry momenta and lines but not at general momenta, as shown in Figure 2a and explored below. The phonon dispersions for other twinning choices are shown in the Supporting Information.

## Weyl Points

We focus on the band crossing points formed by phonon branches 18 and 19 in the frequency range of 20.5–24.0 THz, as they are relatively isolated from the other bands and show the most interesting topological features. On the *q*_*z*_ = 0 plane, no band crossing points are formed between these two phonon branches as the two bands are far away from each other. In contrast, the two phonon branches tend to touch the *q*_*z*_ = 0.5 plane (in units of reciprocal lattice vector 2π/*c*).

Along the A–L line, the two bands form an avoided crossing (i.e., they are gapped), as shown in Figure 2a. The point group along this line is 2*′*, so there is no nontrivial unitary point-group symmetry, and hence there is only one irreducible representation (IRREP), GP_1_, to which both bands belong, as shown in Figure 2a. On the other hand, the point group of the high-symmetry line Q = H–A is 2, which gives rise to two different IRREPs: Q_1_ and Q_2_. We find that bands 18 and 19 belong to different IRREPs along Q and, as such, these bands form a stable Weyl point. By 3-fold rotation and time-reversal symmetry, there are thus a total of six Weyl points on the *q*_*z*_ = ±0.5 plane, all carrying the same Chern number  within the same space group. However, for different space groups, the charges  of the Weyl points on the *q*_*z*_ = ±0.5 plane are opposite, i.e.,  for *P*3_1_21 α-quartz and  for *P*3_2_21 α-quartz, respectively.

In addition to the Weyl points on the *q*_*z*_ = ±0.5 plane, there are also six Weyl points at generic momenta:  for *P*3_1_21 α-quartz at (−0.15, 0.15, −0.49), (0, −0.15, −0.49), (0.15, 0, −0.49), (0, 0.15, 0.49), (0.15, −0.15, 0.49) and (−0.15, 0, 0.49), and  for *P*3_2_21 α-quartz at (−0.15, 0.15, 0.49), (0, −0.15, 0.49), (0.15, 0, 0.49), (0, 0.15, −0.49), (0.15, −0.15, −0.49) and (−0.15, 0, −0.49), respectively (for the choice of unit cells in Figure 1a). The positions of all of the Weyl points in the Brillouin zone are shown in Figure 2b. The six Weyl points at general **q** in a single enantiomorph are related to each other by the 3-fold (nonsymmorphic) rotation symmetries and time reversal, neither of which flip the chirality.

## Surface States

**Figure 3:**
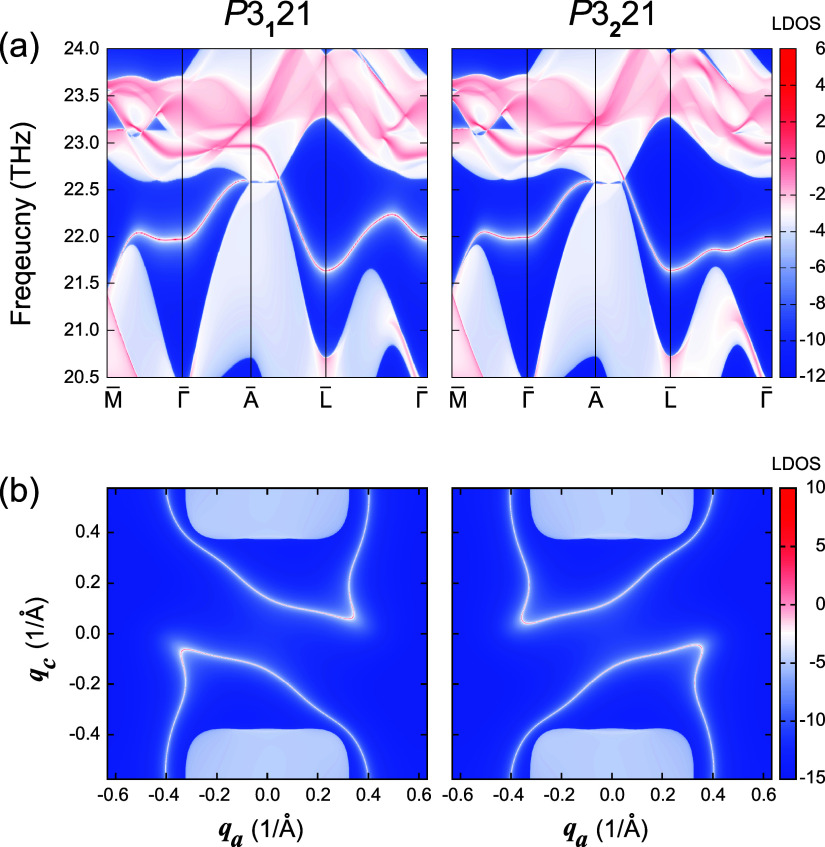
Local density of states (LDOS) of (a) topological surface states on the (010) surface of *P*3_1_21 (No. 152) and *P*3_2_21 (No. 154) α-quartz with the unit cell related by *m*_*z*_ symmetry (Leydolt twinning) and (b) their isofrequency surface arcs at 22.1 THz. Note that the bulk Weyl points are observed at 22.5 THz, so there is no projection of the bulk Weyl points in panel (b).

The surface states of the Weyl points projected on the (010) surface are shown in Figure 3a, for the same choice of unit cell as in Figure 1a. The surface local densities of states (LDOS) correspond to the logarithm of the surface spectrum function *A*(ω, **q**), which is calculated from the imaginary part of the surface Green’s function,^87^ with larger LDOS indicating more surface contributions. The projections of the bulk Weyl points are connected via surface arcs. The surface arcs along the high-symmetry lines M̅–Γ̅–A̅–L̅ are exactly the same for both enantiomorphs for this choice of unit cell, whereas those at generic momenta along the L̅–Γ̅ are different from each other.

To get a better view of the distribution of the surface arcs in the reciprocal space, the isofrequency surface arcs at 22.1 THz are plotted in Figure 3b. The surface arcs at a fixed frequency, with the choice of unit cells shown in Figure 1a for *P*3_1_21 and *P*3_2_21 α-quartz, are related by reflection symmetry along the *q*_*c*_ direction. This is analyzed further below.

## Phonon Dispersion and Twinning

In Nature, quartz crystals frequently form merohedral twins. Crystal twins are regions of two or more adjacent crystals of the same mineral but with differing orientations. The relationship between twins is specified by a twinning operation, which specifies how the regions are mapped to each other. Merohedral twins have parallel lattices, which significantly restricts the possible twinning operations.^94^ Such twins can be of the same chirality if the twinning operation preserves the handedness (e.g., translations, rotations, or screw symmetries), or they form enantiomorphic pairs if the twinning operation changes handedness (e.g., mirror, inversion, rotoinversion, or glide symmetries). In the latter case, the twinning will occur between space groups *P*3_1_21 (No. 152) and *P*3_2_21 (No. 154), which form an enantiomorphic pair.^95,96^

The three most important merohedral twins of quartz are Dauphiné, Brazil, and Leyoldt (also known as combined-law or Liebisch) twins. Of these, Dauphiné twins occur as twinning between two crystals with the same handedness, whereas Brazil and Leydolt twins occur between different enantiomorphs.^94^ Each of these is characterized by a different twinning operation. Dauphiné twinning occurs between two crystals whose crystallographic axes are related by a *C*_2*z*_ symmetry (2-fold rotation around the *c*-axis), and Brazil twinning occurs between crystals related by  (inversion through the origin), whereas Leydolt twinning occurs between crystals related by  (mirroring in the plane normal to the *c*-axis). In Nature, Dauphiné and Brazil twinnings are common, whereas Leydolt twinning is rare.^94^ The effect of these symmetry operations on the bulk phonon dispersion is given by

Dauphiné:
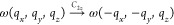
1aBrazil:
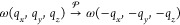
1bLeydolt:
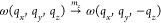
1c

For the standard unit-cell choice in Figure 1a, the crystal structures are related by Leydolt twinning, which explains the relative positions of the Weyl points between the enantiomorphs shown in Figure 2b. As the Berry curvature behaves as a pseudovector, the sign of the Chern number also reverses under mirroring. We next analyze how the bulk and surface phonon band structures behave under twinning.

### Twinned Bulk Band Structures

By definition, none of the twinning operations in eqs 1 are symmetries of the unitary part of the space groups, so we would generically expect different twins to display different band structures. However, we note from Figure 1b (see also Figure S1 in the Supporting Information) that the band structure along high-symmetry lines agrees for all twinning choices.

To explain this, we first note that, in nonmagnetic systems, time-reversal symmetry  enforces ω(**q**) = ω(−**q**). From this, we directly conclude that crystals related by Brazil twinning will display the same bulk dispersion for all **q**. However, this conclusion does not hold for Dauphiné and Leydolt twinning, and we therefore expect the bulk band structure for these twins to generically disagree. To understand the band structure along high-symmetry lines, we start by noting that both Dauphiné twinning and Leydolt twinning lead to the same constraints on the dispersion as, by time-reversal symmetry, ω(−*q*_*x*_, −*q*_*y*_, *q*_*z*_) = ω(*q*_*x*_, *q*_*y*_, −*q*_*z*_). It therefore suffices to analyze Leydolt twinning. We see immediately from eqs 1 that, on the *q*_*z*_ = 0 and *q*_*z*_ = 0.5 planes, all twinning types will display the same dispersion. Thus, the only high-symmetry lines left to discuss are Δ = A*′*–Γ–A and P = H*′*–K–H. Along both of these lines, 2_110_ symmetry (2-fold rotation with respect to the [110] axis) guarantees that the dispersion is symmetric around Γ or K, respectively, and therefore transforming *q*_*z*_ → −*q*_*z*_ does not change the dispersion. Thus, the band structures look identical at all high-symmetry points and along all high-symmetry lines for all twins, as confirmed in Figure S1 in the Supporting Information. However, this is not true at generic momenta, as shown in Figure 2a. We note in passing that the lower-symmetry chiral space-group pairs *P*3_1_ and *P*3_2_ do not have 2_110_ rotation symmetry, so that in crystals belonging to this chiral space-group pair (twinned by the same operations), we would expect the dispersions to disagree even along the high-symmetry line P (Δ remains symmetric around Γ by time-reversal symmetry).

### Twinned Surface States

**Figure 4:**
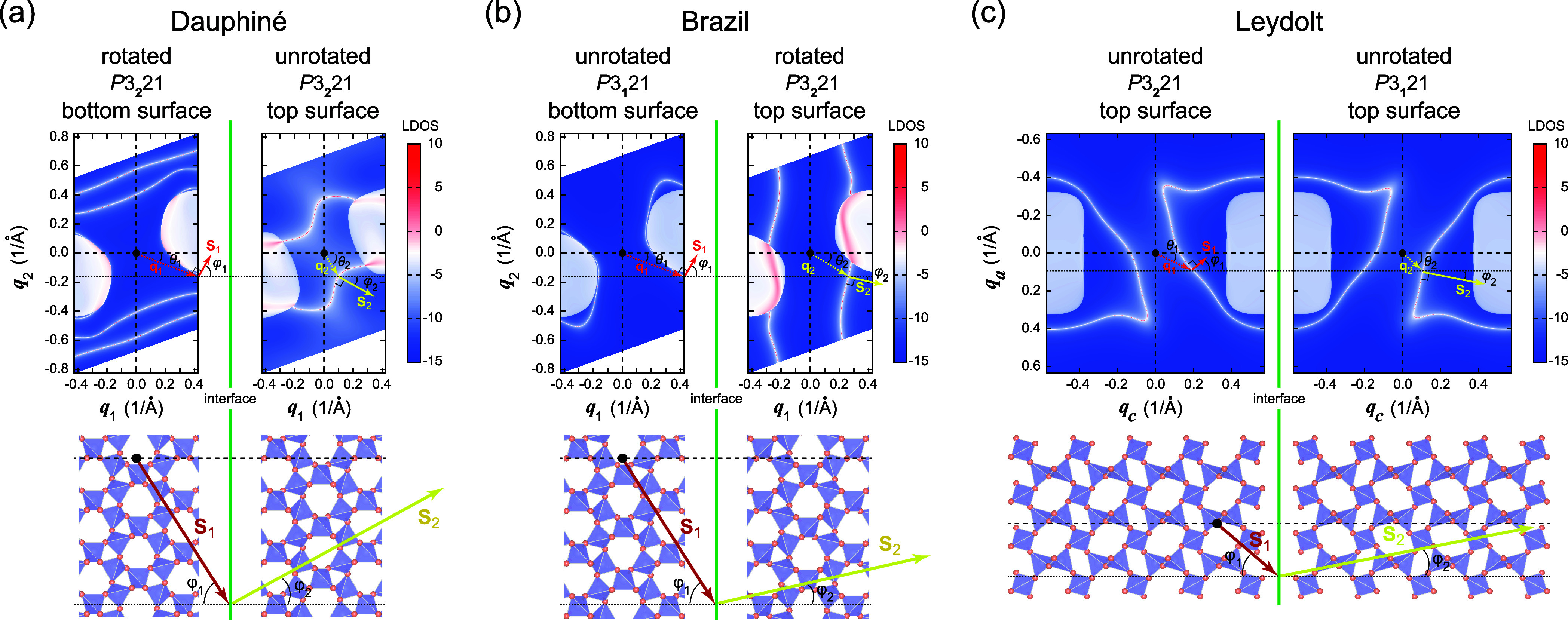
Negative refraction of surface Weyl arcs for (a) Dauphiné twinned quartz on the (101) surface at 22.3 THz between the bottom and top surfaces of rotated and unrotated *P*3_2_21 α-quartz, respectively (related by a *C*_2*z*_ rotation), (b) Brazil twinned quartz on the (101) surface at 22.3 THz between the bottom and top surfaces of unrotated *P*3_1_21 and rotated *P*3_2_21 α-quartz, respectively (related by an inversion operation), and (c) Leydolt twinned quartz on the (010) surface at 22.1 THz between the top surfaces of *P*3_2_21 and *P*3_1_21 α-quartz (related by a *m*_*z*_ mirroring operation). The rotated crystal structures in panels (a) and (b) are obtained by rotating the original crystal structures in Figure 1a by *C*_2*z*_.

The relationship between the surface states for the different crystal twins is more subtle, as this depends on both the crystal termination and the surface symmetries. In what follows, we consider only contact twinning, where the twins form straight twin boundaries. In this case, the crystal termination and surface symmetries are determined by the choice of contact plane. For Dauphiné growth twins and Brazil twins, the contact plane between the twin structures frequently features long straight segments, most commonly oriented along the (101) crystal face.^94^ For Leydolt twinning, not much is known about the twin boundaries. We plot the surface phonons for Dauphiné, Brazil, and Leydolt twinning in Figure 4a–c. For Dauphiné and Brazil twinning, we choose a realistic (101) surface and plot the top and bottom surface, respectively, to represent a stacking of twins. Since not much is known about Leydolt twinning, we consider a conceptually illuminating case by plotting the top surface of the (010) plane for both enantiomorphs. This removes complications due to surface terminations and enables a straightforward analysis of the relationship of the surface bands to chirality and topology.

For Dauphiné and Brazil twinning with realistic surface termination, there is no clear relationship between the isofrequency contours, as shown in Figures 4a and 4b. This is because of the complicated interplay between surface termination and twinning operations. Nonetheless, negative refraction is possible as discussed below. For Leydolt twinned quartz, we find, as shown in Figures 3a and 4c, that the surface band structures agree along the high-symmetry lines but differ at generic points. This may be a result of remnant time-reversal symmetry, or of the antiunitary symmetry , which leaves *q*_*y*_ invariant. We show the surface band structures for the same (010) termination for all twinning operations in Figure S2 in the Supporting Information. In general, it is nontrivial to predict the surface symmetries, as these will be influenced by surface termination and reconstruction. However, the surface arcs shown in Figure 4c arise as a consequence of the bulk Weyl points, and should therefore display some topological protection. We expect these surface states to curve oppositely in the different enantiomorphs, as they are related by a handedness-inverting symmetry, flipping the chirality. Such an inversion of the direction in the surface arcs can give rise to negative refraction at the surface of twins.

## Negative Refraction

We plot the schematics of negative refraction of surface Weyl arcs for all three twin types in Figures 4a–c. For surface phonons with wave vector **q**_1_ and incidence angle θ_1_, the tangential wave vector is conserved,^5^ i.e., **q**_1_ sin θ_1_ = **q**_2_ sin θ_2_ (where **q**_2_ and θ_2_ are the wave vector and refraction angle), exhibiting positive refraction for the wave vector. However, the Poynting vector **S**, which is normal to the isofrequency surface arcs and directed along the energy flow, can exhibit negative refraction, as demonstrated by the Poynting vectors **S**_1_ and **S**_2_ with incidence and refraction angles of φ_1_ and φ_2_, respectively. As a result of negative refraction, Poynting vectors **S**_1_ and **S**_2_ of the surface phonons are on the same side of the interface normal.

Although negative refraction can occur in all three twin types, the open shape of the isofrequency contours for Dauphiné and Brazil twinned quartz shown in Figures 4a,b, despite supporting negative refraction, arises due to a complicated interplay of the surface terminations and twinning operation. On the other hand, in Leydolt twinned crystals, benefiting from the isofrequency contours of *P*3_1_21 and *P*3_2_21 α-quartz related by chirality and topology, we generically expect negative refraction at the interface. Interestingly, the negative refraction of Leydolt twinning is tunable by varying the surface phonon frequency, as shown in Figure S3 in the Supporting Information. Such negative refraction can transform the linear interface between surfaces of *P*3_2_21 and *P*3_1_21 α-quartz into a lens capable of focusing/defocusing phonon waves, depending on the frequency-tunable incidence and refraction angles.

In terms of feasibility for synthesizing such interfaces, we note that, in Nature, quartz twin crystals are much more abundant than the untwinned ones,^64^ and in virtually every natural quartz, the two morphologically distinct natural crystals are internally twinned.^65,66^ Moreover, the interfaces are experimentally realizable, as it has been reported that an atomically sharp internal interface between two enantiomorphs can be synthesized.^97^ This technique may also enable the systematic growth of simpler interfaces of quartz, such as the (010) surface of the Leydolt twinned quartz studied above.

In terms of measuring negative refraction, previous experiments have used an illumination frequency of ∼27 THz by fabricating a gold antenna on one side of the interface to measure the propagation wave,^5^ which is similar to the frequency in our work. The refractive behavior can be measured by a tunable quantum cascade laser in a scattering-type scanning near-field optical microscope.^98,99^

In terms of potential applications, negative refraction in the terahertz region holds significant implications for thermal emission by controlling the flow of the thermal energy. This discovery can also facilitate the development of thermal imaging techniques for medical imaging, aerospace, and manufacturing.

In summary, we explore the relationship between chiral crystal structures, twinning, and topological charges of Weyl points. We find that, depending on the choice of twinning operation, the bulk band structure for different twins can agree or disagree at generic points. Furthermore, we find that the Weyl points in opposite chiral structures carry opposite Chern numbers, which can lead to negative refraction at the twinning surface between different enantiomorphs.

## Supporting Information Available

Bulk phonon dispersions for four different choices of unit cell, corresponding to different twinning choices (Figure S1); (010) surface arcs at 22.2 THz for various choices of unit cell for α-quartz, corresponding to various twin operations (Figure S2); topological surface arcs at 22.1 and 22.2 THz on the (010) surface of *P*3_1_21 (No. 152) and *P*3_2_21 (No. 154) α-quartz related by *m*_*z*_ symmetry (Leydolt twinning) (Figure S3) (PDF)
