# Supplementary material for: Negative Refraction of Weyl Phonons at Twin Quartz Interfaces
Source: ACS Mater Lett. 2024 Feb 5;6(3):847–55. doi: 10.1021/acsmaterialslett.3c00846 (PMC10915867; doi:10.1021/acsmaterialslett.3c00846)
Supplement: Supplementary file 1 — tz3c00846_si_001.pdf [file tz3c00846_si_001.pdf]

Supporting Information for "Negative refraction of Weyl phonons at twin quartz interfaces"

Gunnar F. Lange,[†,‖] Juan D. F. Pottecher,[‡,‖] Cameron Robey,[¶] Bartomeu Monserrat,[*,†,§] and Bo Peng[*,†]

†*Theory of Condensed Matter Group, Cavendish Laboratory, University of Cambridge, J. J. Thomson Avenue, Cambridge CB3 0HE, United Kingdom*

‡*St Catharine's College, University of Cambridge, Trumpington Street, Cambridge CB2 1RL, United Kingdom*

¶*St John's College, University of Cambridge, St John's Street, Cambridge CB2 1TP, United Kingdom*

§*Department of Materials Science and Metallurgy, University of Cambridge, 27 Charles Babbage Road, Cambridge CB3 0FS, United Kingdom*

‖*These authors contributed equally to this work.*

E-mail: bm418@cam.ac.uk; bp432@cam.ac.uk

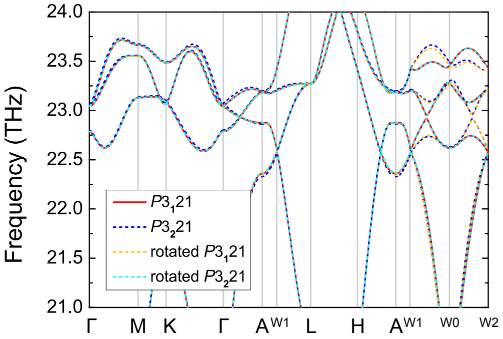

Figure S1: Bulk phonon dispersions for four different choices of unit cell, corresponding to different twinning choices. Starting with $P3_121$, $P3_221$ is generated by a mirror-rotation m_z normal to the c-axis (corresponding to Leydolt twinning). Rotated $P3_121$ is generated from $P3_121$ by a 2_{001} rotation along the c-axis (corresponding to Dauphiné twinning). Similarly, rotated $P3_221$ is generated from $P3_221$ by a 2_{001} rotation along the c-axis (corresponding to Brazil twinning relative to $P3_121$).

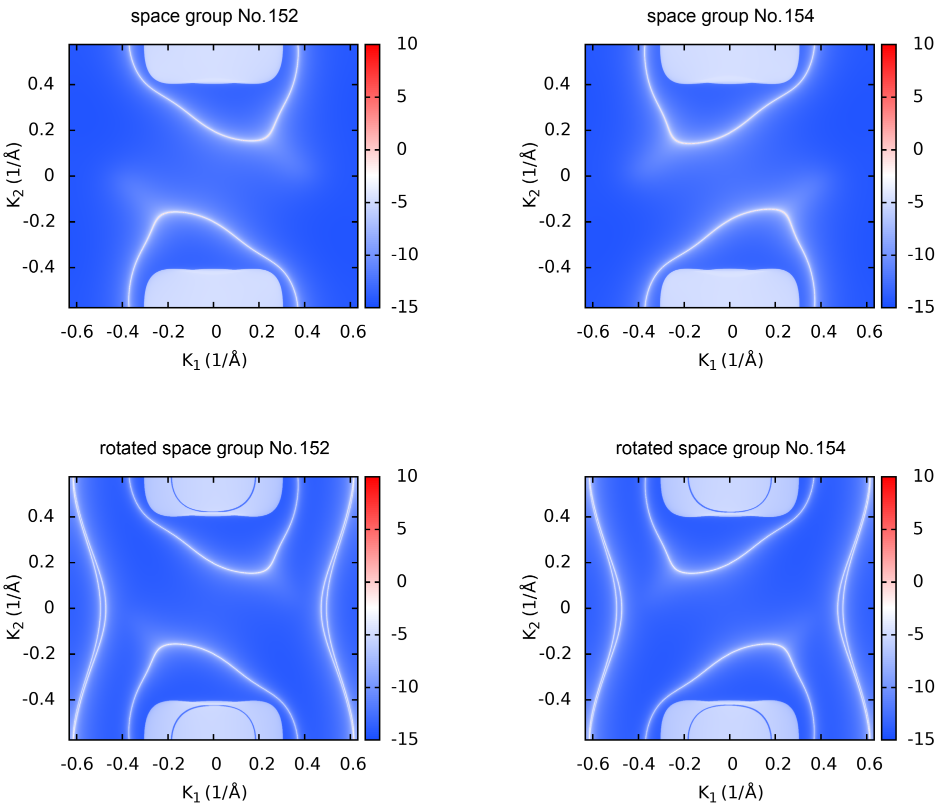

Figure S2: (010) surface arcs at 22.2 THz for various choices of unit cell for α-quartz, corresponding to various twin operations, as described in Fig. S1. The surface state depends on termination, so that additional surface states can appear upon rotation. Crucially, however, different enantiomorphs curve in opposite direction, as can be seen by comparing the two columns.

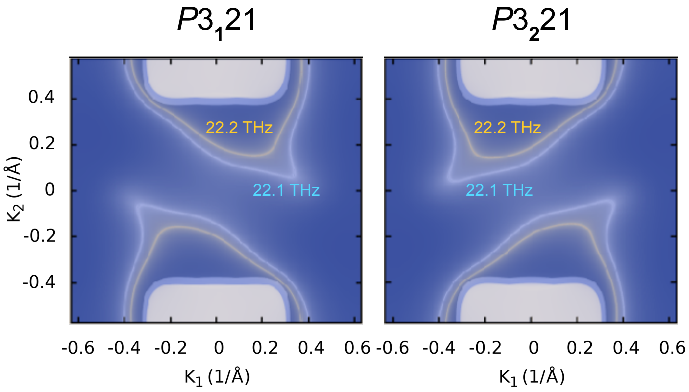

Figure S3: Topological surface arcs at 22.1 and 22.2 THz on the (010) surface of $P3_121$ (No. 152) and $P3_221$ (No. 154) α-quartz related by m_z symmetry (Leydolt twinning).
